# SARS-CoV-2 spike protein accelerates systemic sclerosis by increasing inflammatory cytokines, Th17 cells, and fibrosis

**DOI:** 10.1186/s12950-023-00362-x

**Published:** 2023-12-21

**Authors:** Ha Yeon Jeong, Jin-Sil Park, Jin Seok Woo, Kun Hee Lee, Jeong Won Choi, Hye Yeon Kang, Hyun Sik Na, Yeon Su Lee, In Gyu Um, Sung-Hwan Park, Mi-La Cho

**Affiliations:** 1 The Rheumatism Research Center, Catholic Research Institute of Medical Science, College of Medicine, The Catholic University of Korea, Seoul, 06591 Korea; 2 Lab of Translational ImmunoMedicine, Catholic Research Institute of Medical Science, College of Medicine, The Catholic University of Korea, Seoul, 06591 Korea; 3 Department of Biomedicine & Health Sciences, College of Medicine, The Catholic University of Korea, Seoul, 06591 Korea; 4 Department of Medical Life Sciences, College of Medicine, The Catholic University of Korea, Seoul, 06591 Korea; 5 Division of Rheumatology, Department of Internal Medicine, Seoul St. Mary’s Hospital, College of Medicine, The Catholic University of Korea, Seoul, 06591 Korea

**Keywords:** Coronavirus Disease 2019 (COVID-19), Autoimmune Disease, Systemic sclerosis, Inflammation, Fibrosis

## Abstract

**Background:**

Coronavirus disease 2019 (COVID-19) induces a dysfunctional immune response, inflammation, autoantibody production, and coagulopathy, which are symptoms that bear resemblance to those of autoimmune diseases, including systemic sclerosis (SSc).

**Methods:**

While there is a single case report suggesting an association between COVID-19 and SSc, the effects of COVID-19 on SSc are not yet fully understood. Human embryonic kidney 293 (HEK293) cells were transfected with the SARS-CoV-2 spike protein gene, in the presence of TGF-β. The expression levels of fibrosis-related proteins were measured via Western blotting. A bleomycin (BLM)-induced SSc mouse model was employed, wherein mice were injected with the gene encoding the SARS-CoV-2 spike protein and the ACE2 receptor. The levels of fibrosis, autoantibodies, thrombotic factors, and inflammatory cytokines in tissues and serum were analyzed.

**Results:**

In vitro, the expression levels of fibrosis marker proteins were elevated in the spike protein group compared to the control group. In vivo, the skin thickness of SSc mice increased following exposure to the SARS-CoV-2 spike protein. Furthermore, the levels of autoantibodies and thrombotic factors, such as anti-phospholipid antibodies (APLA), were significantly increased in the presence of the protein. Flow cytometry analysis revealed increased expression of the proinflammatory cytokine IL-17 in the skin, lungs, and blood. Moreover, tissue fibrosis and levels of inflammatory cytokines in skin and lung tissues were markedly escalated in SSc mice subjected to the protein.

**Conclusion:**

COVID-19 may accelerate the development and progression of SSc by intensifying fibrosis through the upregulation of inflammation, autoantibody production, and thrombosis.

**Supplementary Information:**

The online version contains supplementary material available at 10.1186/s12950-023-00362-x.

## Background

The coronavirus disease 2019 (COVID-19) pandemic, which is caused by severe acute respiratory syndrome coronavirus 2 (SARS-CoV-2), has had a profoundly devastating impact on global healthcare, societal well-being, and economies [1, 2]. The primary target of COVID-19 is the respiratory tract, leading to lung complications, including pneumonia [3]. Within the pulmonary tissues, COVID-19 instigates hyperinflammation, characterized by the excessive activation of immune cells, which in turn results in the uncontrolled secretion of inflammatory cytokines. This excessive response, known as a cytokine storm, can culminate in multi-organ failure and death [4, 5]. Notably, patients with autoimmune diseases are considered at heightened risk for COVID-19 infection and severe clinical manifestations, largely attributed to their compromised immune systems from the usage of immunosuppressive drugs, steroids, or anti-cytokine medications [6–8]. This has spurred researchers to investigate the relationship between COVID-19 and autoimmune diseases. In a meta-analysis of 62 observational studies, patients with autoimmune diseases including rheumatic diseases and inflammatory bowel disease had a higher prevalence of COVID-19 compared to the general population [6].

SSc, also known as scleroderma, is a multifaceted autoimmune connective tissue disease characterized by extensive vasculopathy, dysregulation of the immune system, and excessive fibrosis affecting the skin and internal organs [9]. Early in the progression of SSc, vascular changes cause impaired vasoconstriction and angiogenesis, while immune aberrations lead to the production of autoantibodies and the release of inflammatory cytokines and chemokines [10, 11]. These factors contribute to increased vascular permeability and the migration of immune cells into the extracellular matrix, culminating in pathological fibrosis and organ dysfunction [10, 12].

Pulmonary fibrosis, also referred to as interstitial lung disease (ILD), is a common pulmonary manifestation in SSc patients and is a leading cause of SSc-related mortality [13]. There is no selective and fully effective treatment for SSc-related ILD; however, immunosuppressants such as cyclophosphamide or rituximab decelerate the progression of lung disease and are predominantly used in patient management [14, 15]. Given that COVID-19 affects the lungs and individuals on immunosuppressive medication are particularly susceptible to infection, it can be postulated that COVID-19 infection could be detrimental to SSc patients [6–8].

Among Brazilian SSc patients with SSc-ILD, patients with moderate-to-severe COVID-19 had a higher incidence of ILD (64.1%), PAH (20%), and cardiac involvement (27.5%) compared to patients with mild COVID-19 [16]. There have even been reports that healthy people without a history of SSc developed clinical symptoms of SSc after infection with COVID-19. A case report was published showing that a man in good health with no special medical history developed clinical symptoms suggestive of SSc, including skin rush, periorbital edema and conjunctivitis, Raynaud’s phenomenon, and early stages of ILD after COVID-19 infection [18]. Additionally, a case report was reported in which a woman with a history of anxiety and depression developed symptoms of abnormal nailfold capillaries, sclerodactyly of the fingers, and ILD after suffering from COVID-19 pneumonia and was diagnosed with SSc according to the 2013 ACR-EULAR classification criteria for SSc. [19]. Vaccination against COVID-19 can provide protection against mortality in patients with underlying SSc-associated ILD [17]. However, the mechanisms through which COVID-19 impacts the progression of SSc remain largely unexplored. This study investigated the effects of SARS-CoV-2 on the development of SSc using a bleomycin (BLM)-induced SSc mouse model.

## Methods

### Cell culture and transfection with SARS-CoV-2 spike protein and ACE2 protein

HEK293 were cultured in Dulbecco’s Modified Eagle’s Medium (DMEM, 12,800,017; Gibco), supplemented with 10% (v/v) fetal bovine serum (FBS, #16,000,044; Gibco), and maintained at 37 °C in a 5% CO_2_ atmosphere. The cells were seeded at a density of 3.5 × 10^5^ cells per well in 6-well culture plates and allowed to grow for 20–24 h. Subsequently, the cells were transfected with 2 µg plasmid encoding either SARS-CoV-2 spike protein (pBOB-CAG-SARS-CoV-2-Spike-HA, #12,260; Addgene) or ACE2 protein (pLEX307-ACE2-puro, #158,448; Addgene) using 6 µL X-treme GENE HP DNA transfection reagent (6,366,236,001; Roche), following the manufacturer’s instructions. HEK293 cells overexpressing SARS-CoV-2 spike protein and ACE2 were starved in 1X insulin-Transferrin-Selenium (GIB-51,300–044; Gibco) DMEM for 12 h. Then the cells were stimulated with 10 ng/mL TGF-beta1 for 24 h in 1X ITSA DMEM; 48 h post-transfection, the cells were harvested for Western blotting analysis.

### Animals

Seven-week-old male C57BL/6 mice were procured from Orient Bio Inc. (Seongnam, Korea). The mice were housed under specific-pathogen-free conditions at the Institute of Medical Science of the Catholic University of Korea. They had ad libitum access to water and were provided with standard mouse chow (Ralston Purina, St. Louis, MO, USA). All experimental procedures involving animals were conducted in accordance with the Laboratory Animals Welfare Act, the Guide for the Care and Use of Laboratory Animals, and the Guidelines and Policies for Rodent Experiments as provided by the Institutional Animal Care and Use Committee of the Catholic University of Korea. Furthermore, the procedures conformed to all National Institutes of Health (USA) guidelines (permit number: [2023-0064]). To minimize any distress or suffering, all mice were anesthetized with isoflurane (2–2.5%) and euthanized by cervical dislocation.

### Bleomycin and spike S and ACE2 induction

Bleomycin was administered over a period of 2 weeks. To induce SSc in mice, bleomycin was dissolved in PBS. Male C57BL/6 mice aged 8 weeks were injected subcutaneously with 50 µg bleomycin dissolved in 100 µL PBS daily for 2 weeks. To develop a COVID-19 infection model, mice were intramuscularly injected with 100 µg pLEX307-ACE2-puro (#158,448; Addgene) in 50 µL saline twice every 5 days for 35 days. In addition, 100 µg pcDNA3.1-SARS-2-Spike (#145,032; Addgene) was intramuscularly injected into the Spike group twice every 5 days for 30 days.

### Subcellular fractionation

Proteins in the cells were extracted using RIPA lysis and extraction buffer (Cat. 89,901; Thermo) supplemented with complete protease inhibitor (Cat. 78,438; Thermo) and 0.5 M EDTA (Cat. 78,438; Thermo). After incubating the cocktail solution on ice for 20 min, the samples were centrifuged at 14,000 *g* for 15 min at 4 °C. The supernatants were collected into new tubes. Protein concentrations were determined using the Pierce BCA Protein Assay Kit (Cat. 23,225, Thermo).

### Western blotting

Each sample containing 20 µg protein was electrophoresed on 10% polyacrylamide gradient gels and then transferred to nitrocellulose membranes. The proteins were separated via SDS-PAGE, transferred to Hybond enhanced chemiluminescence (ECL) membranes (10,600,001; Cytiva), and incubated with antibodies against anti-ACE2 (ab108252; Abcam, 1:1,000), anti-2019-nCoV spike (40,591-MM42; Sino Biological Inc., 1:1,000), anti-alpha smooth muscle actin (α-SMA) (ab7817[1A4]; Abcam, 1:1,000), anti-Col1a1 (PA5–89,281; Invitrogen, 1:1,000), and GAPDH (ab181602[EPR16891]; Abcam, 1:2,000) using the SNAP i.d. Protein Detection System (Millipore, Billerica, IL, USA). After incubation with the corresponding secondary anti-mouse (SC-2005; Santa Cruz) and anti-rabbit (SC-2357; Santa Cruz) antibodies, the protein bands were visualized using X-film (100NIF ; EA8EC).

### Enzyme-linked immunosorbent assay (ELISA)

Human SARS-CoV-2 spike protein and mouse anti-phospholipid antibody levels in the serum were measured via ELISA. Serum was isolated from mice injected with or without SARS-CoV-2 spike plasmid DNA. SARS-CoV-2 spike (40,591-MM42; Sino Biological Inc.) and mouse anti-phospholipid antibody (MBS742389; MBS) levels were measured via ELISA according to the manufacturer’s instructions. Absorbance was measured at 405 nm on an ELISA microplate reader (Molecular Devices Inc., Sunnyvale, CA, USA).

### Isolation of spleen, blood, skin, and lung T cells

Splenocytes and blood cells circulating in the vasculature were treated with ammonium-chloride-potassium (ACK) buffer to lyse red blood cells. Single-cell suspensions were incubated with phorbol 12-myristate 13-acetate (P8139; Sigma), ionomycin (I0634; Sigma), and protein transport inhibitor (51–2091KZ; BD) in Roswell Park Memorial Institute (RPMI) 1640 medium supplemented with 5% fetal bovine serum (FBS) (#16,000,044; Gibco) at 37℃ and 5% CO_2_ for 4 h. Then the cells were washed with phosphate buffer solution containing 0.2% bovine serum albumin and 0.02% sodium azide. Skin from the fibrosis-induced area of 5 mice was placed into PBS media containing 5 mM EDTA (EDS-500G; Sigma), 10 mM HEPES (H0396; TCI), and 10% FBS. Skin fragments were incubated on a rotating plate for 30 min in a dry, non-gassed incubator maintained at 37℃. Then the contents were vortexed vigorously for 10 s and strained through a 40 μm strainer (SPL93040; SPL Life Sciences) to collect the tissue. The skin was transferred into a new tube containing digestion media (containing 0.7 mg/mL collagenase IV (17,104,019; Gibco), 0.7 mg/mL DNase I (11,284,932,001; Roche), and 0.7 mg/mL Dispase II (D4693; Sigma)). Then this tube was placed on a rotating plate for 30 min in a dry, non-gassed incubator maintained at 37℃. The contents were vortexed for 10 s and filtered through a 40 μm strainer into a new tube. Lungs with induced fibrosis from 5 mice were placed into a Petri dish (93,060; TPP) and chopped into small pieces using a scalpel. The lung pieces were incubated in a digestion solution (containing 2 mg/mL collagenase IV and 0.1 mg/mL DNase I) for 1 h. Subsequently, 10% DMEM media was added, followed by centrifugation at 2,000 rpm for 5 min. The contents were filtered through a 40 μm strainer into a new tube. Cells were resuspended in 4 mL 40% Percoll in 5% RPMI media. The cell suspension in 40% Percoll was layered over 4 mL 80% Percoll in a new tube and centrifuged at 2,000 rpm for 30 min at 4℃.

### Flow cytometry

For effector T cell analysis, cells were initially stained with PerCP-Cy5.5-conjugated CD4 (45–0042–82; eBioscience) at 4℃ for 30 min and then washed with buffer solution. To stain intracellular cytokines, cells were fixed and permeabilized with a kit (554,715; BD) at 4℃ for 30 min. After washing with buffer solution, the cells were stained with PE-conjugated IL-4 (554,435; BD) and FITC-conjugated IL-17 (11–7177–81; eBioscience) at 4℃ for 30 min. After a final wash with buffer solution, the cells were analyzed by flow cytometry (CytoFLEX; Beckman Coulter).

### Histological assessment

#### Hematoxylin and eosin staining and masson’s trichrome staining

To assess the severity of fibrosis and inflammation, the thickness of the skin dermis was measured after sacrificing the animals. Isolated tissues were fixed in 10% (v/v) neutral-buffered formalin (HT501320; Sigma) and embedded in paraffin. µThe Sect. 5 μm thick were deparaffinized using xylene and dehydrated using an alcohol gradient. The sections were stained with hematoxylin and eosin (H&E) and Masson’s trichrome.

To stain H&E, sections were stained hematoxylin (S2-5; yd-diagnostics) for 5 min. Wash to tap water 3 times. And then for bluing except to nuclear, sections were incubated 1% HCL for 30s. Wash in tap water. For staining cytosol, the sections were stained eosin (#32,002; MUTO). Wash in tap water. The sections were dehydrated using an alcohol gradient and mounting (S3023; DAKO).

To Masson’s trichrome stain (25088-1; Polysciences), For formalin fixed tissue, re-fix in Bouin’s solution (BOU-OT-L; BIOGNOST) at 60℃ for 1 h. wash in flowing water for 5 min. Stain in weigert’s iron hematoxylin for 10 min. Wash in running water for 5 min. Rinse in distilled water. Stain in biecrich scarlet-acid fuchsin solution for 5 min. Rinse in distilled water. Differenctiated in phosphomolybdic-phosphotungstic acid solution for 10 min. Discard solution, stain in aniline blue for 5 min. Rinse in a tap water. Differentiated in 1% acetic acid for 1 min. Wash in tap water. The sections were dehydrated using an alcohol gradient and mounting.

### Immunohistochemistry

After deparaffinization, tissue Sect. 5 μm thick underwent antigen retrieval using proteinase K (S3020; Dako) in Tris-EDTA buffer (93,283; Sigma). Then the sections were incubated with 3% hydrogen peroxide (H0300; Samchun Chemical) in methyl alcohol (59; Duksan) to block endogenous peroxidase activity. Sections were stained with reagents (S0809, K4001, K400311–2, K346811–2, and CS703; Dako) according to the manufacturer’s instructions. Primary antibodies against alpha-SMA (ab7817; Abcam, 1:5,000), Col1a1 (PA5–29,569; Invitrogen, 1:800), IL-6 (NB600–1131; NOVUS, 1:600), IL-4 (PA5-25165; Invitrogen, 1:200), IL-17 A (ab79056; Abcam, 1:600), TNF-alpha (Ab6671; Abcam, 1:150), and IL-1 beta (NB600–633; NOVUS, 1:400) were used for staining.

### Immunofluorescence

Tissue Sect. 5 μm thick were deparaffinized and underwent antigen retrieval using proteinase K (S3020; Dako) in Tris-EDTA buffer (93,283; Sigma). The sections were incubated with 3% hydrogen peroxide (H0300; Samchun Chemical) in methyl alcohol (59; Duksan) to block endogenous peroxidase activity. Sections were stained with primary antibodies, including anti-CD4 (14–9766–82; eBioscience), anti-IL-4 conjugated with PE (12–7041–82; eBioscience), and anti-IL-17 A conjugated with FITC (11–7177–81; eBioscience), in 10% normal goat serum in PBS. In addition, sections were stained with anti-mouse IgG secondary antibody conjugated with APC (A-865; Thermo). Nuclei were stained with DAPI (D35271; Invitrogen) in PBS. All stained sections were imaged using a confocal laser scanning microscope (LSM700).

### Statistical analysis

To analyze histology in skin and lung, the positive area of DAB-stained were assessed in IHC staining using ImageJ software. In confocal staining, the number of fluorescent cells was counted.

The results are presented as the mean ± SEM. Statistical analysis between two groups was performed using an unpaired t-test. Statistical significance was determined using a p-value cut-off of 0.05. GraphPad Prism version 9.50 (GraphPad Software, San Diego, California, USA) for Windows was used to plot the data.

## Results

### SARS-CoV-2 spike protein induces fibrosis in HEK293 cells

**Fig. 1:**
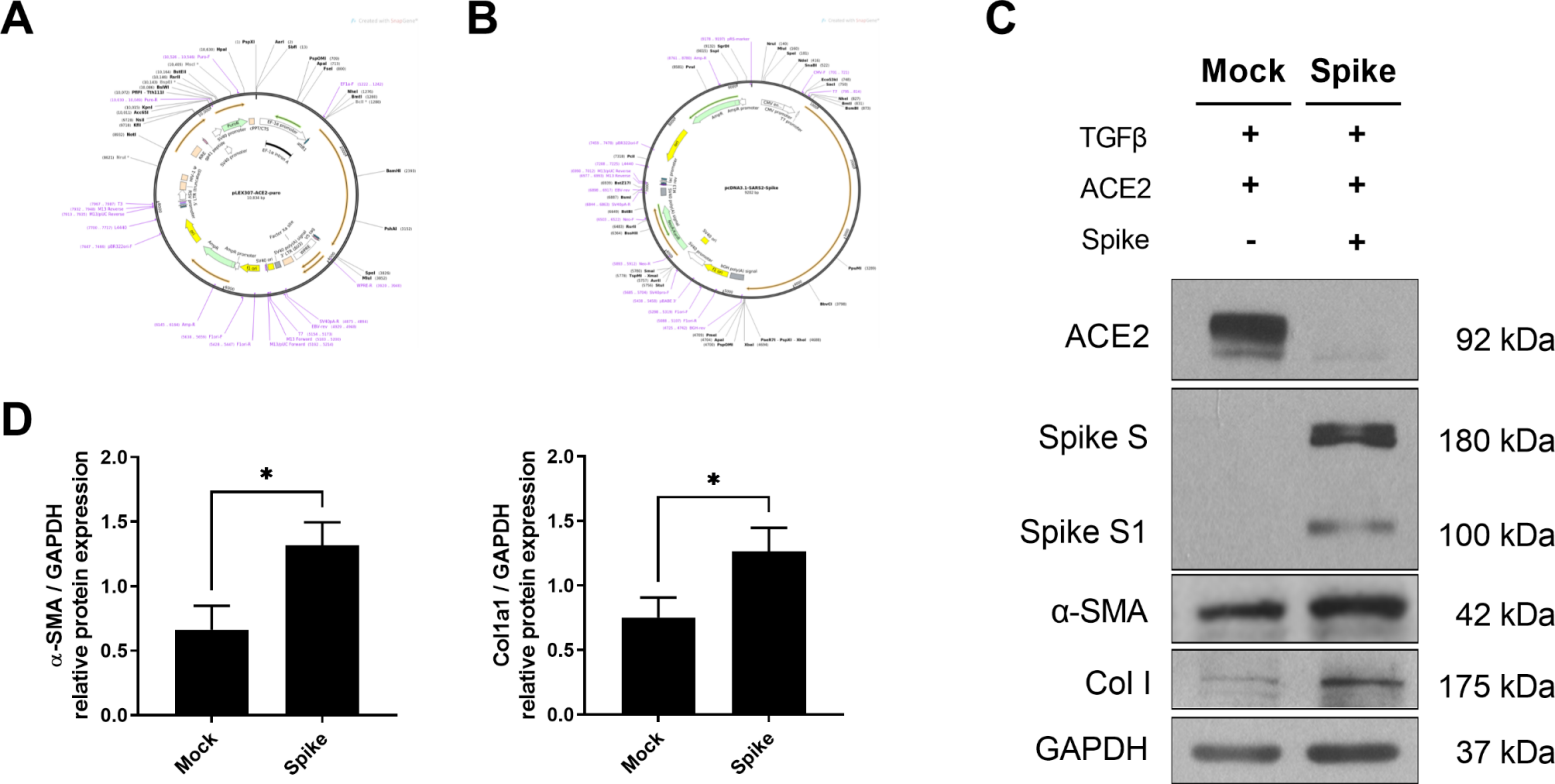
Overexpression of SARS-CoV-2 spike protein induces fibrotic effects in the HEK293 cell line. (**a, b**) Schematic representation and cloning site regions of the pcDNA3.1-SARS2-spike and pLEX307-ACE2-puro plasmids. (**c**) Western blotting analysis detecting the protein levels of fibrosis markers α-SMA and collagen type I with or without the overexpression of pcDNA3.1-SARS2-spike and pLEX307-ACE2-puro in the HEK293 cell line. (**d**) Graph illustrating the levels of α-SMA and collagen type I with or without the overexpression of pcDNA3.1-SARS2-spike and pLEX307-ACE2-puro in the HEK293 cell line. The data were expressed as the mean ± SEM for triplicate experiments, GAPDH was used as an internal control; *p < 0.05

The SARS-CoV-2 virus affects epithelial and endothelial cells in the lung. The spike protein of SARS-CoV-2 is also known to induce fibrosis. We investigated whether the spike protein could induce fibrosis in HEK293 cells, which do not endogenously express ACE2 and are not permissive to the endocytosis of the spike protein. To this end, we transfected HEK293 cells with plasmid DNA encoding the SARS-CoV-2 spike protein and ACE2 (Fig. 1A and B). We initially treated the cells with equal volumes of ACE2 plasmid under two different conditions, with or without the spike protein plasmid (Fig. 1C and Fig. S1). When we confirmed the expression of the ACE2 protein, we observed different levels of ACE2 protein between the cells transfected (or not) with the spike protein. This is because ACE2 expression is regulated by the spike protein, which results in decreased ACE2 expression. Subsequently, we measured the levels of fibrosis markers such as alpha-SMA and Col1a1. These proteins were upregulated by the spike protein (Fig. 1C and D).

### SARS-CoV-2 spike protein aggravates systemic sclerosis in a bleomycin-induced mouse model

**Fig. 2:**
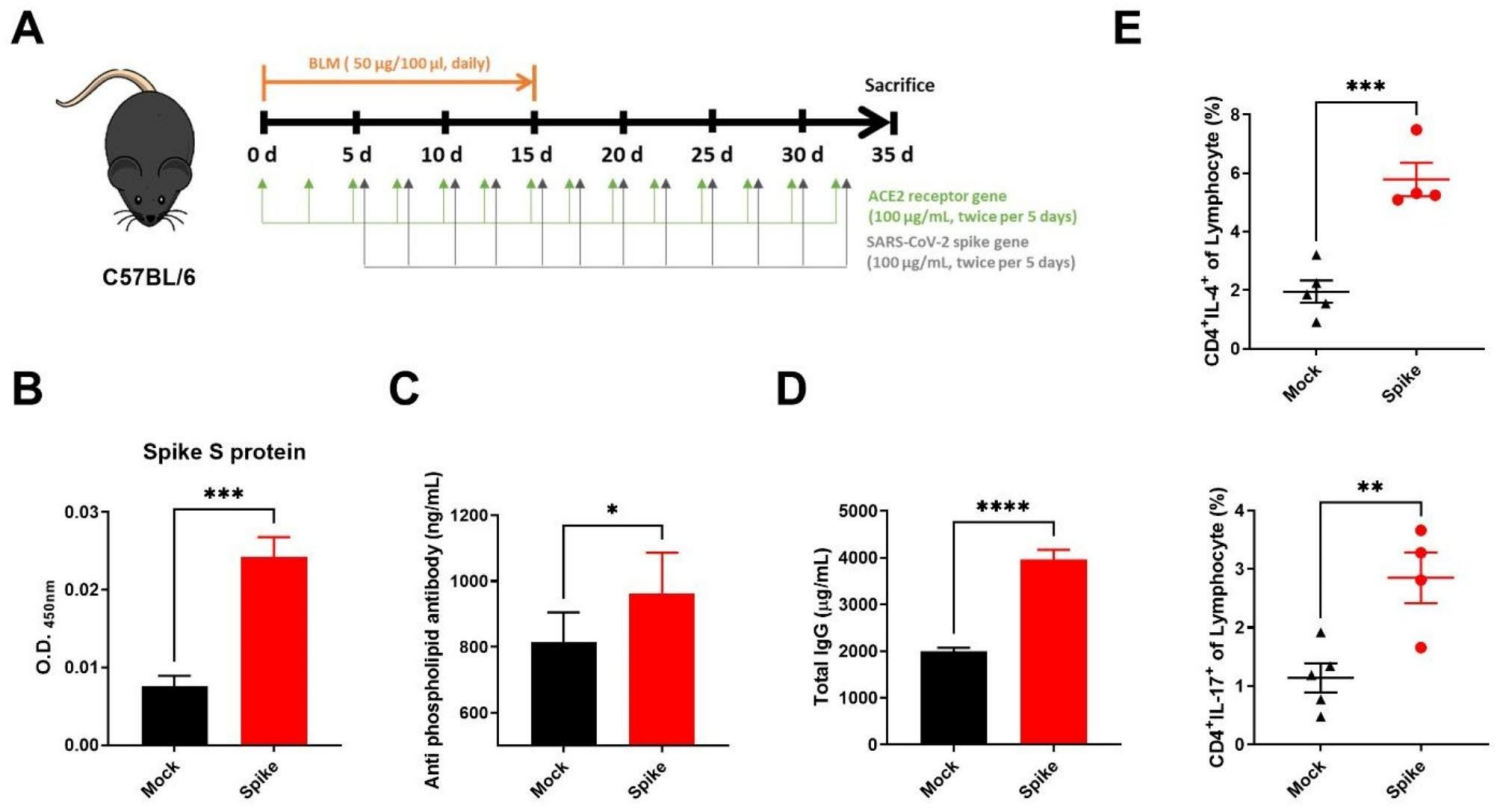
Activation of the immune system in a BLM-induced SSc mouse model following overexpression of pcDNA3.1-SARS2-Spike and pLEX307-ACE2-puro (n = 5). (**a**) Experimental timeline depicting the schedule of pcDNA3.1-SARS2-spike and pLEX307-ACE2-puro overexpression in the BLM-induced SSc mouse model. The mice received 100 µg/mL pcDNA3.1-SARS2-spike and 100 µg/mL pLEX307-ACE2-puro via intramuscular injection twice every 5 days and 0.5 mg/mL bleomycin via subcutaneous injection daily for 2 weeks. (**b–d**) Enzyme-linked immunoassay measurements of spike S protein, anti-phospholipid antibody, and IgG in serum from BLM-induced SSc mice over 35 days. (**e**) Flow cytometric analysis representing the percentage of ex vivo Th2 and Th17 cells from blood in BLM-induced SSc mice

To investigate how the SARS-CoV-2 spike protein affects SSc without causing further infection-related damage in the SSc model, we utilized WT C57BL/6J mice that do not express human ACE2 and are not susceptible to infection by most SARS-CoV-2 strains. We induced skin fibrosis by subcutaneously injecting 50 µg bleomycin for 2 weeks. To mimic SARS-CoV-2 infection, we injected plasmid DNA encoding human ACE2 and the SARS-CoV-2 spike protein intramuscularly into mice (Fig. 2A). Initially, we measured the expression of the spike protein by examining the injected plasmid DNA. We confirmed its presence in the serum of mice injected with it (Fig. 2B). We also observed an increase in anti-phospholipid antibodies, which are autoantibodies in autoimmune diseases that cause thrombosis and vasculopathy (Fig. 2C). Furthermore, we detected an increase in the levels of total IgG in the serum of the group immunized with the protein (Fig. 2D). After 5 weeks, we analyzed the proportions of Th2 and Th17 cells in the peripheral blood via flow cytometry (Fig. 2E). We observed an increase in Th2 and Th17 cells in the group injected with the spike protein, indicating heightened immune system activation and autoimmune symptoms.

### SARS-CoV-2 spike protein contributes to the proliferation of effector T cells

**Fig. 3:**
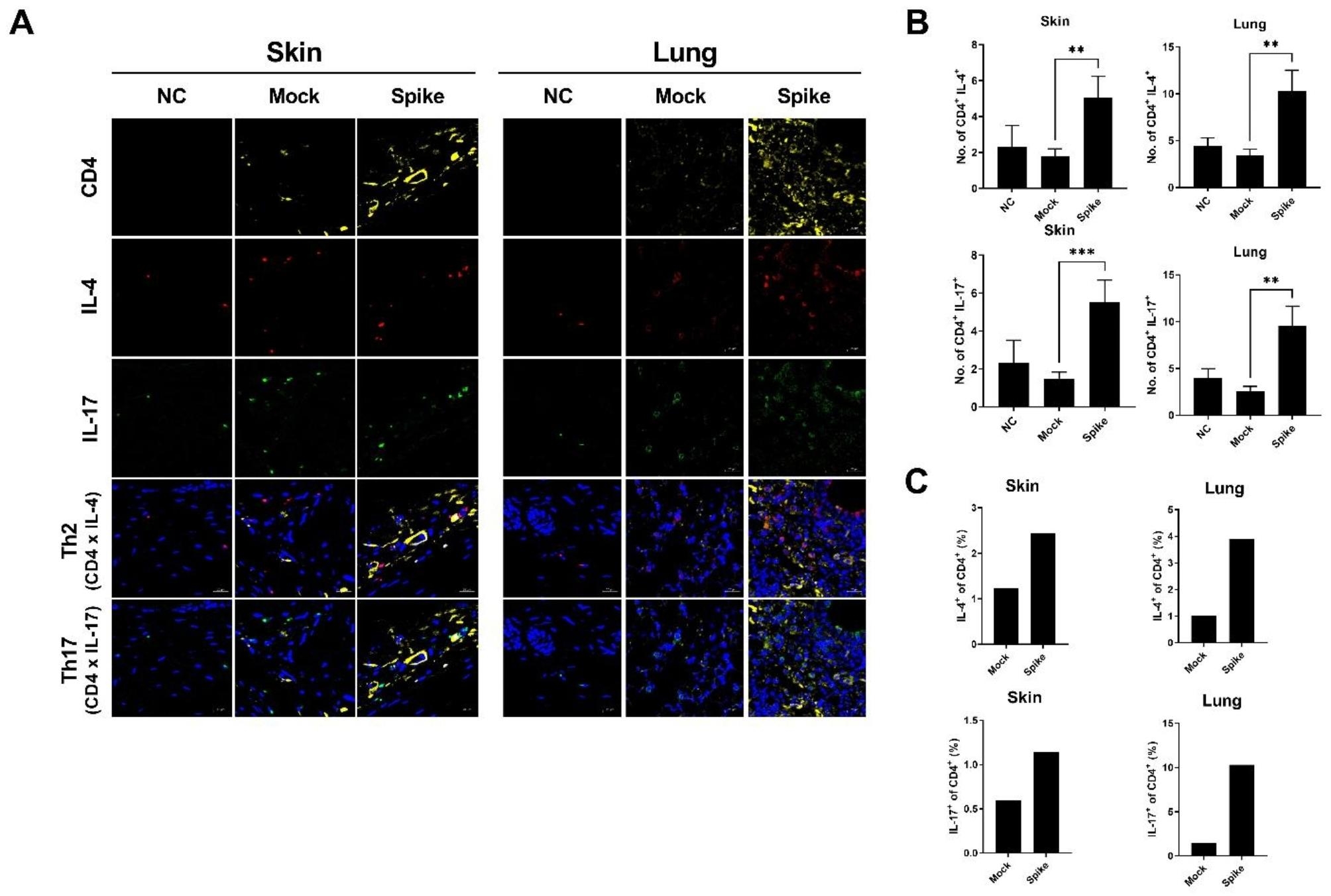
Accelerated fibrosis in BLM-induced SSc mice following injection of pcDNA3.1-SARS2-Spike and pLEX307-ACE2-puro (n = 5). (**a**) Confocal microscopy analysis showing CD4 (FITC), IL-17 (PE), and IL-4 (APC) staining. (**b**) Graph depicting the number of IL-4 + cells and IL-17 + cells among CD4 + positive cells. (**c**) Flow cytometric analysis illustrating the percentage of ex vivo Th2 and Th17 cells from the skin or lung in BLM-induced SSc mice

We hypothesized that the proportions of Th2 and Th17 cells in the skin and lung tissue increase in fibrotic tissues. To detect the infiltration of lymphocytes, we utilized immunofluorescence microscopy (Fig. 3A). The group injected with the SARS-CoV-2 spike protein exhibited increased Th2 and Th17 cell populations in the skin and lung tissues compared to the other group. However, there were no differences between the normal control group and the group without the spike protein (Fig. 3B). We attribute this to the method used to induce the SSc model, where the injection of bleomycin subcutaneously did not induce lung fibrosis as it would have with intratracheal injection. In addition, we analyzed Th2 and Th17 cells in isolated skin and lung tissues from the mouse model. We found that the group injected with the spike protein had a greater increase in the infiltration of Th2 and Th17 cells into the skin and lung tissues compared to controls (Fig. 3C). Based on these results, we concluded that immune cells characteristic of autoimmune responses were activated and infiltrated the damaged tissues due to the presence of the SARS-CoV-2 spike protein in the mouse model.

### SARS-CoV-2 spike protein accelerates fibrosis in skin and lung tissue in SSc mouse model

**Fig. 4:**
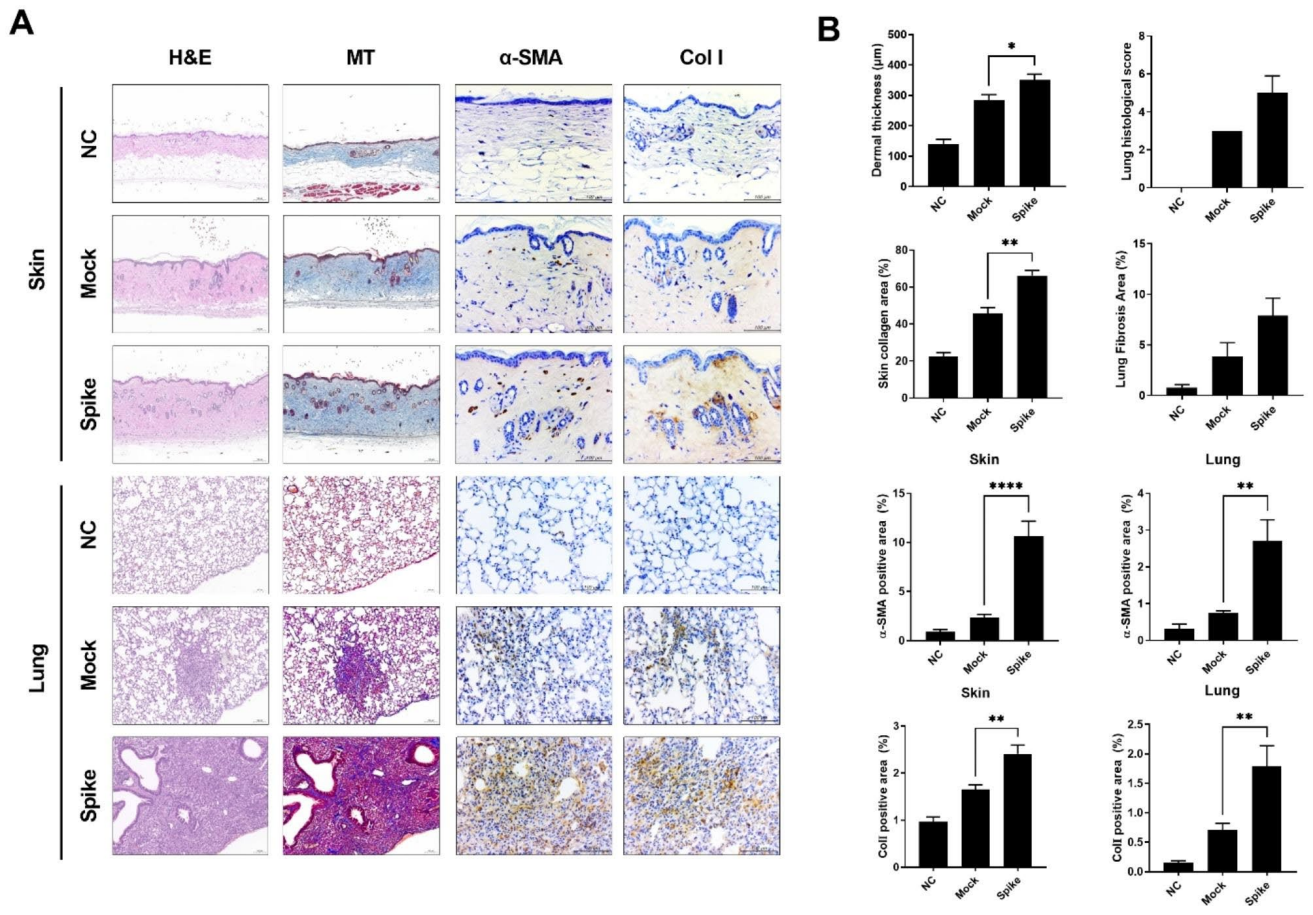
Induction of fibrosis in skin and lung tissues in BLM-Induced SSc mice following injection of pcDNA3.1-SARS2-Spike and pLEX307-ACE2-puro (n = 5). (**a**) Histological analysis of skin and lung tissues isolated from sacrificed SSc mouse models at 5 weeks post-injection, stained with hematoxylin & eosin, Masson’s trichrome, and subjected to immunohistochemistry analysis for α-SMA and collagen type I. (**b**) Graph representing skin dermal thickness, lung histological score, collagen area, α-SMA, and collagen type I positive area in skin and lung tissues

We further hypothesized that the activated effector immune cells could exacerbate the damage to fibrotic tissue. To test this, we assessed the extent of fibrosis using histological staining techniques (Fig. 4A and B). Through H&E staining, Masson’s trichrome staining, and immunohistochemistry, we observed an increase in skin dermis thickness and accumulation of collagen in the skin and lung tissues of the group injected with the SARS-CoV-2 spike protein. In addition, we observed a significant increase in the expression of alpha-SMA and Col1, which are well-known markers for fibrosis, in both skin and lung tissues from the injected group.

### SARS-CoV-2 spike protein increases skin and lung fibrosis in SSc animal model

**Fig. 5:**
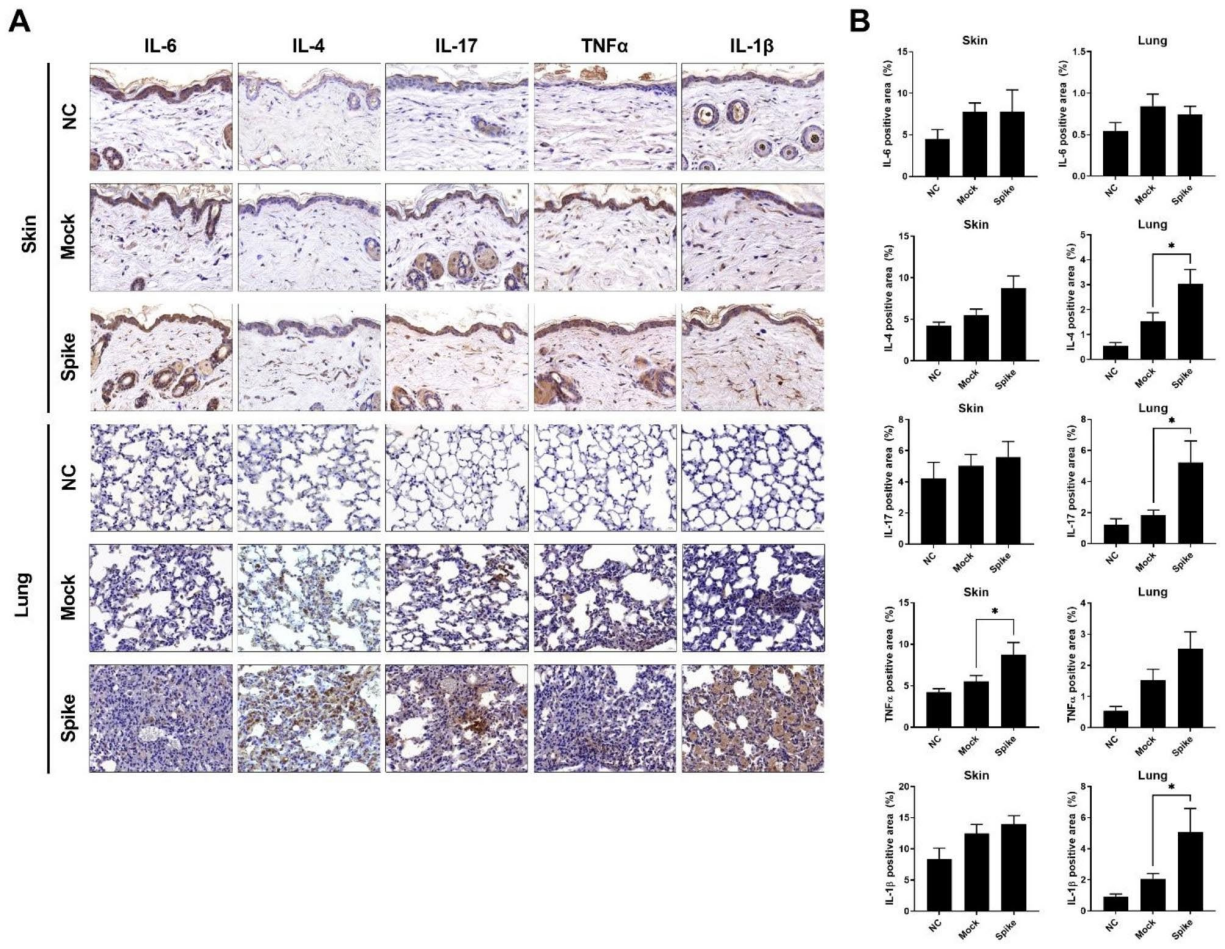
Upregulation of inflammatory cytokines in BLM-induced SSc mice following injection of pcDNA3.1-SARS2-Spike and pLEX307-ACE2-puro (n = 5). (**a**) Immunohistochemical analysis of IL-6, IL-4, IL-17, TNFα, and IL-1β in skin and lung tissues isolated from sacrificed SSc mouse models at 5 weeks post-injection. (**b**) Graph illustrating the area positive for IL-6, IL-4, IL-17, TNFα, and IL-1β in skin and lung tissues

Along with fibrosis, the expression of inflammatory cytokines is also known to increase in SSc. We measured the expression levels of IL-6, IL-4, IL-17, TNF-α, and IL-1β through immunohistochemistry staining (Fig. 5A and B). In skin and lung tissues, the group injected with the spike protein vector displayed a higher increase in inflammatory cytokines compared to the group without the vector. Taken together, these results suggest that the presence of the SARS-CoV-2 spike protein in the tissue environment accelerates both fibrosis and the immune response in SSc.

## Discussion

We explored the impact of the SARS-CoV-2 spike protein on the development and progression of SSc in a murine model. In vitro, overexpression of the SARS-CoV-2 spike protein in the HEK293 cell line, which exhibits epithelial morphology, led to increased production of myofibroblast markers, including α-SMA and collagen type I. In vivo, injection of the SARS-CoV-2 spike protein in BLM-induced SSc mice resulted in the secretion of spike protein and anti-phospholipid antibody, akin to what has been observed in individuals infected with COVID-19. Moreover, there was a notable increase in IgG levels in the serum and the frequency of Th2 and Th17 cells in the blood due to the overexpression of the SARS-CoV-2 spike protein. We also found that the protein led to an increase in inflammatory cytokine-producing cells, particularly Th2 and Th17 cells, in the skin and lung tissues of BLM-induced SSc mice. Importantly, the protein exacerbated collagen accumulation and extracellular matrix deposition in these tissues. Collectively, these findings suggest that COVID-19 might worsen the development of SSc.

The interplay between immune cells and stromal fibroblasts is a critical element in the complex pathogenesis of SSc, which includes autoimmune dysregulation, fibrosis, and vasculopathy [10, 20]. T cells, particularly when excessively activated, have been implicated in the aberrant immune response characteristic of SSc [10, 21]. Our study revealed that the SARS-CoV-2 spike protein increased the infiltration of Th2 and Th17 cells in the skin and lung tissues of BLM-induced SSc mice. Classical T helper 2 (Th2) cells secrete cytokines such as IL-4 and IL-13 and promote fibrosis. Th2 cell profiles have predominantly been observed in skin-infiltrating T cells of SSc patients [22, 23], with elevated levels of IL-4 and IL-13 in serum [24]. Notably, IL-13 levels have been clinically correlated with erythrocyte sedimentation rates, C-reactive protein levels, and the number of nailfold capillaroscopy abnormalities, which are predictors of mortality in patients with SSc [24, 25]. Both IL-4 and IL-13 promote fibroblast proliferation and the production of extracellular matrix proteins, such as collagen [26, 27]. In addition, Th17 cells, which produce IL-17, have more recently been implicated in the pathogenesis of SSc [28]. Elevated levels of IL-17 A and increased frequency of Th17 cells have been observed in the serum and peripheral blood of SSc patients, respectively [29, 30]. The increased Th17 frequency is correlated with disease activity, and Th17-derived IL-17 has been shown to induce fibroblast proliferation and collagen production [31, 32].

SARS-CoV-2 infection elicits a hyperinflammatory response, characterized by the rapid and abundant secretion of inflammatory cytokines [33]. This cytokine storm can affect survival by modulating immune cell functions such as those of macrophages and lymphocytes [33, 34]. Moreover, this inflammatory environment, coupled with intracellular adhesion molecule dysfunction, can increase vascular permeability, facilitating the migration of immune cells into the extracellular matrix [35, 36]. Consequently, the activation of macrophages and CD4 T cells stimulates fibroblasts, leading to their proliferation and the synthesis of collagen and matrix metalloproteinases, which result in fibrosis and potentially internal organ dysfunction [37, 38].

## Conclusions

In conclusion, our study demonstrates that the SARS-CoV-2 spike protein promotes the infiltration of cells expressing inflammatory cytokines, as well as Th2 and Th17 cells, into the skin and lung tissues in a BLM-induced SSc mouse model. In addition, we observed an increase in the expression of fibrotic markers in these tissues. Further investigations are warranted to determine if the SARS-CoV-2 spike protein directly influences the differentiation of T cells into Th2 and Th17 cells, and to elucidate how it affects the interplay between immune cells and fibroblasts. Our findings in the SSc murine model suggest that the SARS-CoV-2 spike protein may expedite the progression of the disease in patients with SSc.

### Electronic supplementary material

Below is the link to the electronic supplementary material.

Supplementary Material 1

## Data Availability

The data that support the findings of this study are available from the corresponding author upon reasonable request.

## Abbreviations

COVID: 19 Coronavirus disease 2019
SARS: CoV-2 Severe acute respiratory syndrome coronavirus 2
SSc: Systemic sclerosis
ILD: Interstitial lung disease
HEK293: Human embryonic kidney 293
TGF: Transforming grouth factor
ACE2: Antiotensin converting enzyme 2
α-SMA: Alpha smooth muscle actin
Col1a1: Collagen type I alpha 1
BLM: Bleomycin
APLA: Anti-phospholipid antibody
IL: Interleukin
TNF: Tumor necrosis factor

## References

[1] Hughes M (2021). Impact of Covid-19 on clinical care and lived experience of systemic sclerosis: an international survey from EURORDIS-Rare Diseases Europe. J Scleroderma Relat Disord.

[2] Hoffmann-Vold AM (2022). Systemic sclerosis in the time of COVID-19. Lancet Rheumatol.

[3] Denton CP (2021). COVID-19 and systemic sclerosis: rising to the challenge of a pandemic. J Scleroderma Relat Disord.

[4] Ragab D (2020). The COVID-19 cytokine Storm; what we know so far. Front Immunol.

[5] Farzi R (2022). The role of antigen-presenting cells in the pathogenesis of COVID-19. Pathol Res Pract.

[6] Akiyama S (2021). Prevalence and clinical outcomes of COVID-19 in patients with autoimmune Diseases: a systematic review and meta-analysis. Ann Rheum Dis.

[7] Li J (2021). COVID-19 Illness and autoimmune Diseases: recent insights. Inflamm Res.

[8] Hamidi Z (2023). A comprehensive review of COVID-19 symptoms and treatments in the setting of autoimmune Diseases. Virol J.

[9] Hughes M, Herrick AL (2019). Systemic sclerosis. Br J Hosp Med (Lond).

[10] Katsumoto TR, Whitfield ML, Connolly MK (2011). The pathogenesis of systemic sclerosis. Annu Rev Pathol.

[11] Pattanaik D (2015). Pathogenesis of systemic sclerosis. Front Immunol.

[12] Gabrielli A, Avvedimento EV, Krieg T (2009). Scleroderma N Engl J Med.

[13] Steen VD, Medsger TA (2007). Changes in causes of death in systemic sclerosis, 1972–2002. Ann Rheum Dis.

[14] Airo P (2004). Intravenous cyclophosphamide therapy for systemic sclerosis. A single-center experience and review of the literature with pooled analysis of lung function test results. Clin Exp Rheumatol.

[15] Sircar G (2018). Intravenous cyclophosphamide vs rituximab for the treatment of early diffuse scleroderma lung Disease: open label, randomized, controlled trial. Rheumatology (Oxford).

[16] de Oliveira SM (2022). Severity and mortality of COVID-19 in patients with systemic sclerosis: a Brazilian multicenter study. Semin Arthritis Rheum.

[17] Panopoulos S (2023). COVID-19 and protection of vaccination in patients with systemic sclerosis-associated interstitial lung Disease. J Scleroderma Relat Disord.

[18] Fineschi S, Report C (2021). Systemic sclerosis after Covid-19 Infection. Front Immunol.

[19] Chandra A, Kahaleh B (2022). Systemic sclerosis (SSc) after COVID-19: a Case Report. Cureus.

[20] Charles C, Clements P, Furst DE (2006). Systemic sclerosis: hypothesis-driven treatment strategies. Lancet.

[21] Jin W, Zheng Y, Zhu P (2022). T cell abnormalities in systemic sclerosis. Autoimmun Rev.

[22] Mavalia C (1997). Type 2 helper T-cell predominance and high CD30 expression in systemic sclerosis. Am J Pathol.

[23] Chizzolini C (2003). Systemic sclerosis Th2 cells inhibit collagen production by dermal fibroblasts via membrane-associated Tumor necrosis factor alpha. Arthritis Rheum.

[24] Hasegawa M (1997). Elevated serum levels of interleukin 4 (IL-4), IL-10, and IL-13 in patients with systemic sclerosis. J Rheumatol.

[25] Riccieri V (2003). Interleukin-13 in systemic sclerosis: relationship to nailfold capillaroscopy abnormalities. Clin Rheumatol.

[26] McGaha TL (2003). Molecular mechanisms of interleukin-4-induced up-regulation of type I collagen gene expression in murine fibroblasts. Arthritis Rheum.

[27] Huang XL (2015). Role of anti-inflammatory cytokines IL-4 and IL-13 in systemic sclerosis. Inflamm Res.

[28] Chizzolini C, Dufour AM, Brembilla NC (2018). Is there a role for IL-17 in the pathogenesis of systemic sclerosis?. Immunol Lett.

[29] Kurasawa K (2000). Increased interleukin-17 production in patients with systemic sclerosis. Arthritis Rheum.

[30] Truchetet ME (2011). Increased frequency of circulating Th22 in addition to Th17 and Th2 lymphocytes in systemic sclerosis: association with interstitial lung Disease. Arthritis Res Ther.

[31] Yang X (2014). Increased frequency of Th17 cells in systemic sclerosis is related to Disease activity and collagen overproduction. Arthritis Res Ther.

[32] Liu M (2014). Interleukin-17A promotes functional activation of systemic sclerosis patient-derived dermal vascular smooth muscle cells by extracellular-regulated protein kinases signalling pathway. Arthritis Res Ther.

[33] Montazersaheb S (2022). COVID-19 Infection: an overview on cytokine Storm and related interventions. Virol J.

[34] McGonagle D (2020). The role of cytokines including Interleukin-6 in COVID-19 induced Pneumonia and Macrophage Activation Syndrome-Like Disease. Autoimmun Rev.

[35] Di Benedetto P (2019). The vessels contribute to fibrosis in systemic sclerosis. Isr Med Assoc J.

[36] Carroll M, Nagarajah V, Campbell S. Systemic sclerosis following COVID-19 Infection with recurrent corticosteroid-induced scleroderma renal crisis. BMJ Case Rep, 2023. 16(3). 10.1136/bcr-2022-253735 PMC10030551 36931690

[37] Wynn TA, Barron L (2010). Macrophages: master regulators of inflammation and fibrosis. Semin Liver Dis.

[38] Zhang M, Zhang S (2020). T cells in Fibrosis and Fibrotic Diseases. Front Immunol.

