# Supplementary material for: SARS-CoV-2 spike protein accelerates systemic sclerosis by increasing inflammatory cytokines, Th17 cells, and fibrosis
Source: J Inflamm (Lond). 2023 Dec 21;20:46. doi: 10.1186/s12950-023-00362-x (PMC10740237; doi:10.1186/s12950-023-00362-x)

Original article

SARS-CoV-2 Spike Protein Accelerates Systemic Sclerosis by Increasing Inflammatory Cytokines, Th17 Cells, and Fibrosis

Ha Yeon Jeong[1,2,3+], Jin-Sil Park[1,2,4+], Jin Seok Woo[1,2], Kun Hee Lee[1,2,3,4], Jeong Won Choi[1,2], Hye Yeon Kang[1,2,3], Hyun Sik Na[1,2,3], Yeon Su Lee[1,2,3], In Gyu Um[1,2,3], Sung-Hwan Park[1,5*], Mi-La Cho[1,2,3,4*]

[1]The Rheumatism Research Center, Catholic Research Institute of Medical Science, College of Medicine, The Catholic University of Korea, Seoul 06591, Korea

[2]Lab of Translational ImmunoMedicine, Catholic Research Institute of Medical Science, College of Medicine, The Catholic University of Korea, Seoul 06591, Korea

[3]Department of Biomedicine & Health Sciences, College of Medicine, The Catholic University of Korea, Seoul 06591, Korea

[4]Department of Medical Life Sciences, College of Medicine, The Catholic University of Korea, Seoul 06591, Korea

[5]Division of Rheumatology, Department of Internal Medicine, Seoul St. Mary's Hospital, College of Medicine, The Catholic University of Korea, Seoul 06591, Korea

Supplementary Fig 1. Unprocessed original images of western blots on Fig 1.

Fig. 1c : ACE2 - 92 kDa

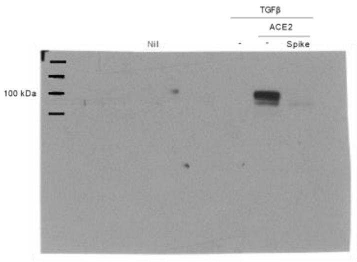

Fig. 1c : Spike - 180-200 kDa

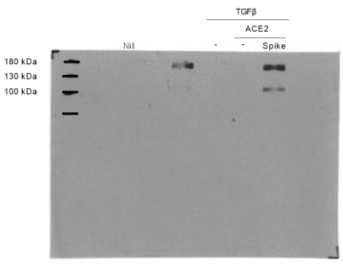

Fig. 1c : Col1a1 - 175 kDa

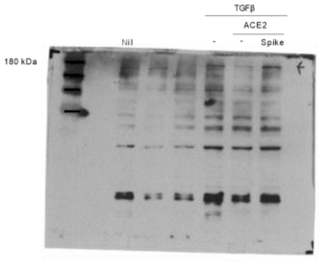

Fig. 1c : GAPDH - 37 kDa

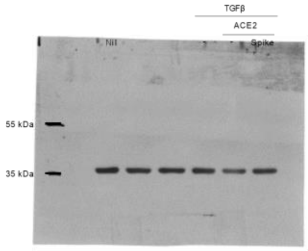

Fig. 1c : α-SMA - 42 kDa

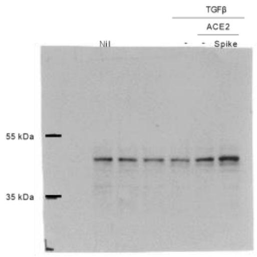

GAPDH - 37 kDa

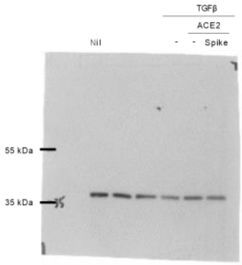

Supplement: Supplementary file 1 — Supplementary Material 1 [file 12950_2023_362_MOESM1_ESM.pdf]
